# Bioavailability of ibuprofen following oral administration of standard ibuprofen, sodium ibuprofen or ibuprofen acid incorporating poloxamer in healthy volunteers

**DOI:** 10.1186/1472-6904-9-19

**Published:** 2009-12-04

**Authors:** Peter M Dewland, Sandie Reader, Phillip Berry

**Affiliations:** 1 Simbec Research, Merthyr Tydfil, CF48 4DR, UK; 2 Reckitt Benckiser Healthcare UK, Dansom Lane, Hull, HU8 7DS, UK

## Abstract

**Background:**

The aim of this study was to compare the pharmacokinetic properties of sodium ibuprofen and ibuprofen acid incorporating poloxamer with standard ibuprofen acid tablets.

**Methods:**

Twenty-two healthy volunteers were enrolled into this randomised, single-dose, 3-way crossover, open-label, single-centre, pharmacokinetic study. After 14 hours' fasting, participants received a single dose of 2 × 200 mg ibuprofen acid tablets (standard ibuprofen), 2 × 256 mg ibuprofen sodium dihydrate tablets (sodium ibuprofen; each equivalent to 200 mg ibuprofen acid) and 2 × 200 mg ibuprofen acid incorporating 60 mg poloxamer 407 (ibuprofen/poloxamer). A washout period of 2-7 days separated consecutive dosing days. On each of the 3 treatment days, blood samples were collected post dose for pharmacokinetic analyses and any adverse events recorded. Plasma concentration of ibuprofen was assessed using a liquid chromatographic-mass spectrometry procedure in negative ion mode. A standard statistical ANOVA model, appropriate for bioequivalence studies, was used and ratios of 90% confidence intervals (CIs) were calculated.

**Results:**

T_max _for sodium ibuprofen was less than half that of standard ibuprofen (median 35 min vs 90 min, respectively; *P *= 0.0002) and C_max _was significantly higher (41.47 μg/mL vs 31.88 μg/mL; ratio test/reference = 130.06%, 90% CI 118.86-142.32%). Ibuprofen/poloxamer was bioequivalent to the standard ibuprofen formulation, despite its T_max _being on average 20 minutes shorter than standard ibuprofen (median 75 mins vs 90 mins, respectively; *P *= 0.1913), as the ratio of test/reference = 110.48% (CI 100.96-120.89%), which fell within the 80-125% limit of the CPMP and FDA guidelines for bioequivalence. The overall extent of absorption was similar for the three formulations, which were all well tolerated.

**Conclusion:**

In terms of T_max_, ibuprofen formulated as a sodium salt was absorbed twice as quickly as from standard ibuprofen acid. The addition of poloxamer to ibuprofen acid did not significantly affect absorption.

## Background

Ibuprofen [(±)-(R, S)-2-4(4-isobutylphenyl)-propionic acid], a chiral 2-arylpropionic acid derivative non-steroidal anti-inflammatory drug, is a widely used and well-tolerated analgesic [1]. Although ibuprofen is a non-selective cyclooxygenase (COX) inhibitor, inhibiting both COX-1 and COX-2 forms, its analgesic, antipyretic and anti-inflammatory effects are achieved principally through COX-2 inhibition [2].

With most analgesics, including ibuprofen, the initial rise in plasma concentration following oral administration is a key factor in determining the time to onset of pain relief [3]. Ibuprofen is rapidly absorbed, and both peak plasma concentrations and maximal analgesic onset are achieved within 1.5-2 hours of oral administration [4].

Key pharmacokinetic studies have demonstrated that there is a linear dose-response relationship between the amount of drug administered and the area under the serum concentration-time curve (AUC) following single doses of ibuprofen (200-800 mg) [4]. There is also a significant correlation between plasma ibuprofen levels and the resultant degree of pain relief, particularly 1 hour after administration [5].

For orally administered standard ibuprofen formulations, it is desirable to have a formulation with a rapid rate of absorption because this is required for rapid pain relief [6]. It is well documented that ibuprofen salts, such as ibuprofen lysine and ibuprofen arginate, are more rapidly absorbed than formulations of free ibuprofen acid [7,8]. Another ibuprofen salt, sodium ibuprofen dihydrate (ibuprofen sodium), manufactured by Hoffmann-La Roche Ltd, was recently shown to be bioequivalent to the lysine and arginate salt forms. Several studies have shown that faster-absorbed formulations lead to faster onset of analgesia [9-14].

Reckitt Benckiser Healthcare International (Hull, UK) has also developed ibuprofen formulations that are expected to be absorbed faster than standard ibuprofen acid. The first is a sodium ibuprofen formulation containing 256 mg of ibuprofen sodium dihydrate per tablet (equivalent to 200 mg ibuprofen acid). The second test ibuprofen formulation contains 200 mg ibuprofen acid plus 60 mg of the surfactant poloxamer 407 (from the poloxamer family of polymeric non-ionic surface active agents) to increase the rate of dissolution of the tablet and therefore enable more rapid absorption. Poloxamers have been used previously to enhance dissolution and bioavailability of poorly water-soluble drugs, including ibuprofen [15].

The aim of this phase I study was to compare the pharmacokinetic properties of a single dose (2 tablets) of each of the two test formulations with those of standard ibuprofen (2 × 200 mg tablets) in healthy volunteers, in order to add to the growing body of evidence for the faster absorption rates of alternative ibuprofen formulations.

This study was conducted in accordance with the Declaration of Helsinki [16], as referenced in EU Directive 2001/20/EC3 [17] and complies with International Conference on Harmonisation, Good Clinical Practice and applicable regulatory requirements.

## Methods

The study protocol, together with participant information and consent documents, were reviewed and approved by South East Wales Local Research Ethics Committee.

### Participants

Healthy male and female volunteers 18-50 years of age (mean 27.2 years) and with a body mass index (BMI) of 20-27 kg/m^2 ^(mean 24.1 kg/m^2^) were included in the study. Participants had provided written informed consent.

Participants with a history of significant disease, any condition that might have interfered with the absorption, distribution, metabolism or excretion of the drugs, or a history of allergy or migraine were ineligible for the study. Those with a history of gastrointestinal disorders, cardiac disease or hypertension, or of psychotic illness were ineligible. Participants reporting frequent dyspepsia, smoking in the previous 6 months and those with a history of drug misuse (including alcohol) were excluded from the study. Participants who had ingested a prescribed drug at any time within the 14 days preceding study enrolment (excluding hormonal contraceptives and hormone replacement therapy) or an over-the-counter (OTC) preparation within 7 days preceding enrolment were also excluded, as were those who had donated blood in the 12 weeks preceding enrolment.

Further exclusion criteria included: known risk factors for AIDS or known HIV positive status, a positive viral serology screen; those with clinically significant abnormal laboratory values at screening; women of childbearing potential, who were pregnant or lactating, seeking pregnancy or failing to take adequate contraceptive precautions; those unable in the opinion of the investigator to comply fully with the study requirements; those previously randomised into the study; previous randomisation in the study or participation in a clinical trial in the previous 12 weeks; citizens of the United States of America and participants of any nationality who were not resident in the UK at the time of the study.

### Study design

This study was a randomised, 3-way crossover, open-label, single-centre, pharmacokinetic study to compare the rate of absorption (assessed from pharmacokinetic parameters) of one standard and two test formulations of ibuprofen given in single doses.

Participants received a single dose of 2 × 200 mg ibuprofen acid tablets (standard ibuprofen; Nurofen^® ^[Reckitt Benckiser Healthcare, UK]), 2 × 256 mg ibuprofen sodium dihydrate tablets (sodium ibuprofen; each equivalent to 200 mg ibuprofen acid) and 2 × 200 mg ibuprofen acid tablets each incorporating 60 mg poloxamer 407 (ibuprofen/poloxamer). A washout period of 2-7 days separated consecutive dosing days.

Participants attended an initial screening visit followed by three treatment visits, each of which required the participants to stay overnight and fast for 14 hours. A post-study follow-up visit occurred 2-7 days after the final treatment visit.

Alcohol consumption was limited to two units/day in the 7 days before the screening visit and participants were required not to drink alcohol from 48 hours before admission to the end of each treatment visit (i.e. until after the 12-hour blood sample). Caffeine intake was prohibited during the study.

### Assessments

A serum pregnancy test was conducted at the initial screening visit and at each treatment visit if appropriate. A urine sample was collected at each of the five visits for (1) urinalysis at the screening and post-study follow-up visits and (2) for drugs of misuse and alcohol at the pre-study visit and at each treatment visit. Haematology and biochemistry assessments were conducted at the screening visit and the post-study visit. On the three treatment days, blood samples (5 ml) were collected from all participants at 5, 10, 15, 20, 25, 30, 35, 40, 50 minutes and 1, 1.25, 1.5, 1.75, 2, 3, 6, 9 and 12 hours post dose for pharmacokinetic analyses. More frequent sampling was conducted in the first hour to obtain accurate pharmacokinetic data during the initial absorption phase.

Safety was assessed in terms of the overall number of participants with adverse events. Adverse events were recorded in the case report forms by the investigator or designee after asking participants "Have you experienced any symptoms or complaints?" before dosing, at 4 and 12 hours post dose, prior to the subject leaving the unit, and again when the subject returned to the unit for their next visit. Spontaneously reported adverse events were also recorded, and laboratory values and vital signs were monitored.

### Plasma ibuprofen determination

Plasma ibuprofen determinations were performed by Simbec Research Limited using a liquid chromatographic-mass spectrometry procedure (LC-MS) in negative ion mode. This procedure was fully validated over the calibration range 0.5-100 μg/ml and a limit of quantitation set at 0.5 μg/ml. Volumes of 0.2 ml of test, standard and quality control sample were spiked with 50 μg flurbiprofen internal standard (i.e. 250 μg/ml) in acetonitrile to facilitate protein precipitation from the plasma. After centrifugation, the supernatant was evaporated to dryness and reconstituted in assay mobile phase. LC-MS analysis of extract was performed on a 5 μm C18 column (5 cm × 0.21 cm) with mass detection by a PE-Sciex API 150EX single quadrupole mass spectrometer under the following conditions:

Mobile Phase:    0.01 M ammonium acetate (49.95% v/v): acetonitrile (49.95% v/v): glacial acetic acid (0.10% v/v)

Flow Rate:    0.3 ml/min

Split Ratio:    1:5

Data acquisition and integration were achieved using a Macintosh computer system running PE Sciex Masschrom version 1.1.1 data handling software incorporating MACQUAN version 1.6 software.

### Study endpoints

The primary endpoints were the pharmacokinetic variables T_max_, C_max_, AUC_0-t _and AUC_0-inf _(Table 1) derived from the plasma ibuprofen concentrations for the three formulations. As a secondary pharmacokinetic endpoint, the cumulative AUCs at each blood sampling time point up to 1 hour after dosing were compared in order to fully explore any differences in the rate of drug absorption between the three formulations.

**Table 1:** Pharmacokinetic variables and their definitions

T_max_	The time to the first occurrence of the maximum plasma concentration
**C_max_**	The observed maximum plasma concentration

**AUC_0-t_**	AUC to the last measurable plasma concentration (Cp), calculated by the linear trapezoidal rule

**AUC_0-inf_**	AUC calculated by linear trapezoidal rule to the last measurable Cp with additional area calculated from Cp/Kel. Kel is the elimination rate constant calculated from the slope of the terminal portion of the plasma profile calculated by least-squares regression of log (concentration) against time

### Sample size determination

The sample size of this study was based on previous work with other similarly fast-absorbed ibuprofen formulations in which the within-subject coefficient of variation for ibuprofen C_max _was found to be 20%. Therefore, using the method of Diletti *et al*. [18], a sample size of 20 subjects was considered sufficient to detect a 20% difference between the test and reference formulations with a power of 80% and alpha of 5%, based upon a test versus reference ratio of 1.05. Since the main parameter of interest in this study was T_max_, the sample size calculation was also based on T_max_. The within-subject standard deviation for T_max _in the previous studies was found to be approximately 70 minutes. In order to detect a difference of 40 minutes in T_max _between the test and reference formulations, it was calculated that a sample size of 20 subjects would be required to give a power of 85%. Furthermore, the sample size of this study was in line with that used in similar published studies [19].

### Statistical analyses

T_max _was analysed using a Wilcoxon matched pairs test. A 95% non-parametric confidence interval (CI) was constructed for the median difference in the T_max _values based on the Hodges-Lehmann estimates.

For the cumulative AUC, AUC_0-t_, AUC_0-inf _and C_max _data, analysis of variance, including the terms for sequence, subject nested within sequence, period and formulation, was carried out on logarithmically transformed data. Point estimates and 90% CIs for the difference between each test treatment and the reference treatment were constructed and then back transformed to give estimates of the ratio of the geometric least squares means and 90% CIs for the ratios.

## Results

### Demographics

A total of 22 participants (15 males and 7 females) qualified for enrolment and were randomised to treatment. The mean age of all participants was 27.2 years and the mean BMI was 24.1 kg/m^2^. No participants withdrew from the study.

### Pharmacokinetic endpoints

**Figure 1:**
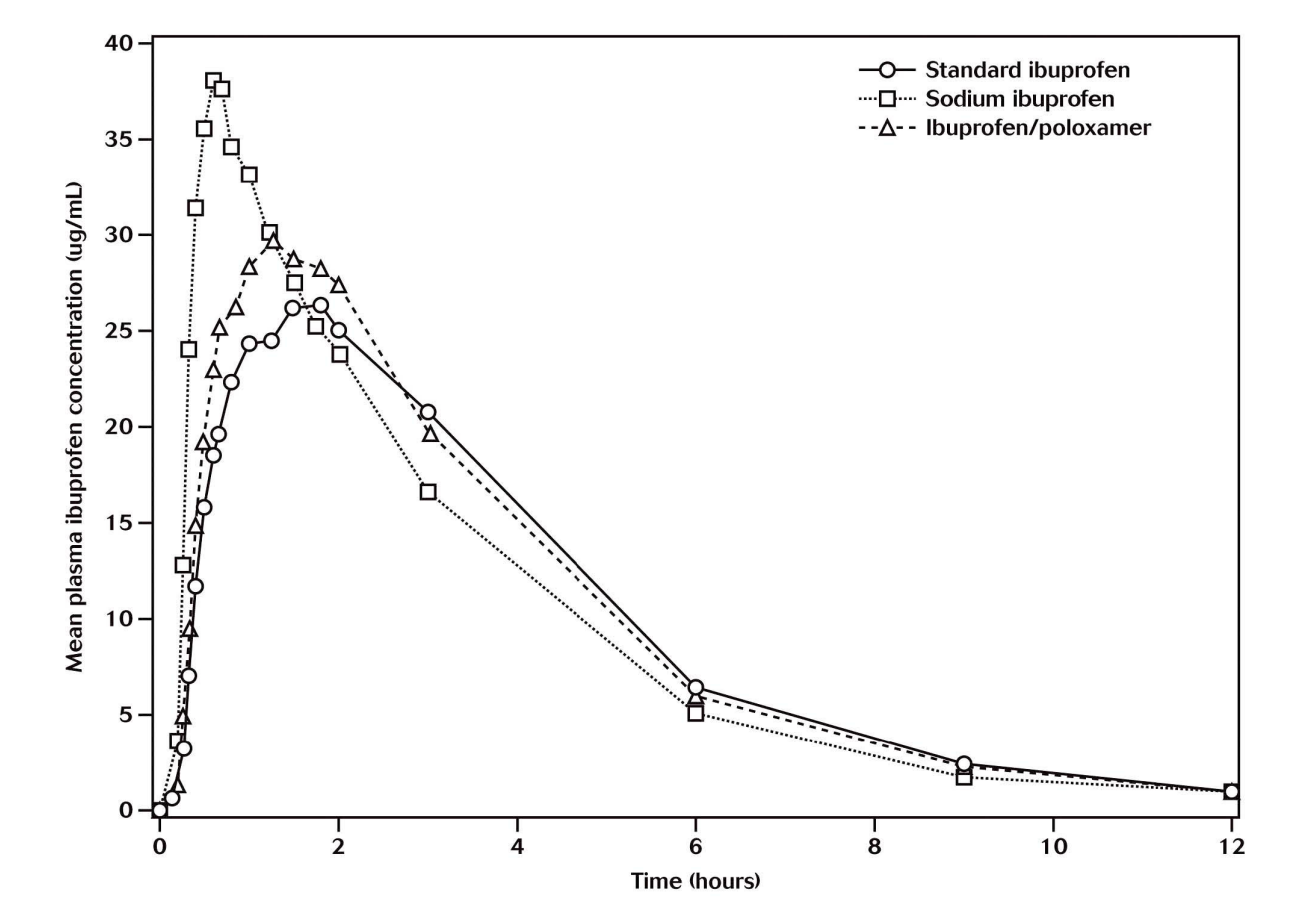
**Mean plasma ibuprofen concentrations (μg/mL)**.

Figure 1 shows the mean plasma concentrations of the two test and one reference ibuprofen formulations. The mean values for C_max_, AUC_0-t _and AUC_0-inf _and the median values for T_max _are presented in Table 2. Administration of sodium ibuprofen resulted in a significantly shorter time to peak plasma concentration (T_max_), compared with standard ibuprofen (median 35 min vs 90 min; *P *< 0.0002). T_max _for sodium ibuprofen was on average 55 minutes shorter than with standard ibuprofen.

**Table 2:** C_max_, AUC_0-t_, AUC_0-inf _and T_max _data

	Sodium ibuprofen (n = 22)	Ibuprofen/poloxamer (n = 22)	Standard ibuprofen (n = 22)
**C_max _(μg/mL)**			
Arithmetic mean	43.01	36.06	32.84
Geometric LS mean	41.47	35.22	31.88

**AUC_0-t _(μg/mL/h)**			
Arithmetic mean	120.57	124.10	119.10
Geometric LS mean	117.79	120.55	115.28

**AUC_0-inf _(μg/mL/h)**			
Arithmetic mean	122.88	126.72	121.99
Geometric LS mean	119.73	122.75	117.71

**T_max _(min)**			
Median	35	75	90

LS: least-squares

Although the T_max _for ibuprofen/poloxamer was on average 20 minutes shorter than that of the standard ibuprofen formulation, this difference was not statistically significant (median 75 min vs 90 min; *P *= 0.1913). The power in terms of T_max _for a *P*-value of 0.0002 was >99% and for a *P*-value of 0.1913 was 31%.

The C_max _results obtained for the sodium ibuprofen formulation and the standard ibuprofen formulation were 41.47 and 31.88 μg/mL, respectively (geometric LS means). The ratio of the test/reference was 130.06% with 90% CIs of 118.86-142.32%. The C_max _obtained for the ibuprofen/poloxamer tablets was 35.22 μg/mL. The ratio of the test/reference was 110.48% with 90% CIs of 100.96-120.89%.

For overall extent of absorption, all three formulations were equivalent, with test/standard formulation ratios of both AUC_0-inf _and AUC_0-t _very close to 100%. The AUC_0-t _values were 117.79 μg/h/mL, 120.55 μg/h/mL and 115.28 μg/h/mL for sodium ibuprofen, ibuprofen/poloxamer and standard ibuprofen, respectively. The AUC_0-inf _values were 119.73 μg/h/mL, 122.75 μg/h/mL and 117.71 μg/h/mL for sodium ibuprofen, ibuprofen/poloxamer and standard ibuprofen, respectively.

**Figure 2:**
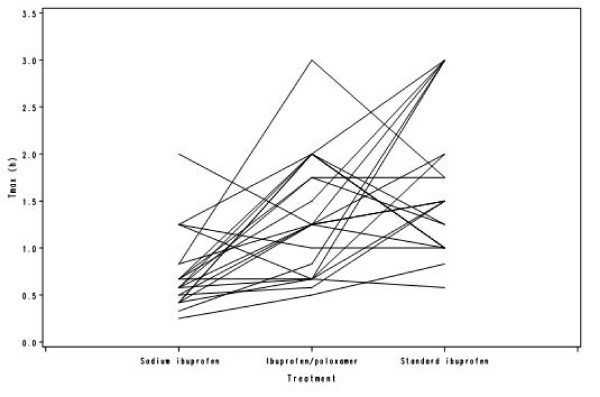
**Spaghetti plot of T_max_**.

**Figure 3:**
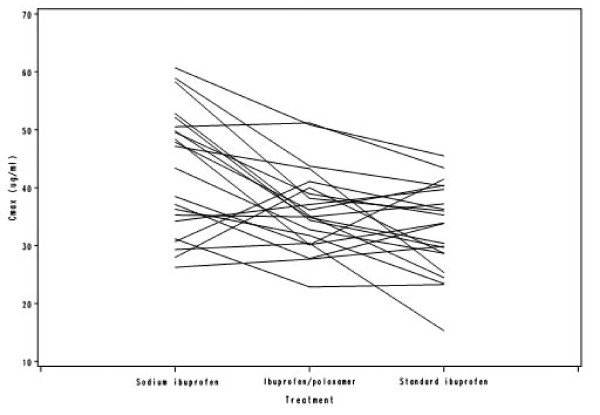
**Spaghetti plot of C_max_**.

**Figure 4:**
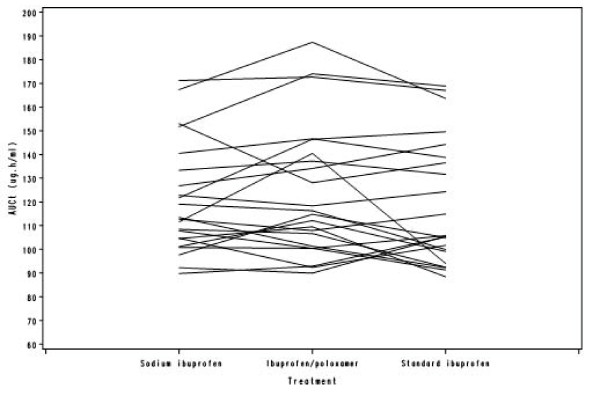
**Spaghetti plot of AUC_0-t_**.

**Figure 5:**
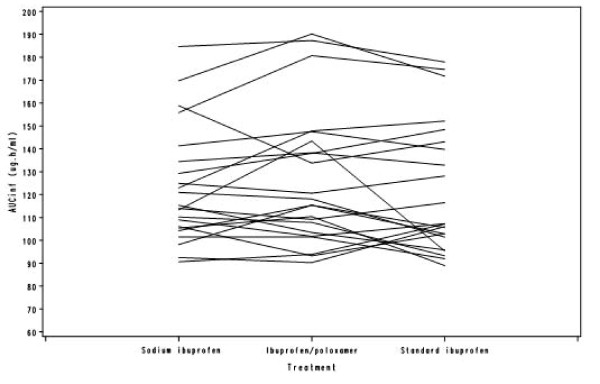
**Spaghetti plot of AUC_0-inf_**.

Table 3 shows the ratios of the sodium ibuprofen/standard formulations and the ibuprofen/poloxamer/standard formulations for partial AUC. Throughout the first hour post dose, the partial AUCs for both the sodium ibuprofen and ibuprofen/poloxamer formulations were higher than those for the standard ibuprofen formulation, with the greatest test/standard formulation absorption ratio occurring at 20 minutes for both test formulations (Table 3). The spaghetti plots provide individual subject information per treatment, for each of the four derived pharmacokinetic parameters (Figures 2, 3, 4 and 5). In general, C_max _was found to be higher and T_max _was lower for sodium ibuprofen than ibuprofen/poloxamer or standard ibuprofen (Figures 2, 3, 4 and 5).

**Table 3:** Statistical analyses of partial AUC data

	Sodium ibuprofen (geometric means)	Ibuprofen/ poloxamer (geometric means)	Standard ibuprofen (geometric means)	Ratio (%) sodium ibuprofen/ standard ibuprofen	90% confidence interval	Ratio (%) ibuprofen/ poloxamer/ standard ibuprofen	90% confidence interval
AUC 0-0.08 (μg/h/mL)	Not estimated	Not estimated	Not estimated	Not estimated	Not estimated	Not estimated	Not estimated
AUC 0-0.17 (μg/h/mL)	0.06	0.03	0.03	217.32	95.29-495.60	118.65	49.21-286.08
AUC 0-0.25 (μg/h/mL)	0.49	0.13	0.09	529.77	262.01-1071.16	138.56	68.53-280.17
AUC 0-0.33 (μg/h/mL)	1.61	0.50	0.18	905.93	462.33-1775.19	278.80	142.28-546.31
AUC 0-0.42 (μg/h/mL)	3.57	1.24	0.55	644.02	362.66-1143.69	224.33	126.32-398.37
AUC 0-0.50 (μg/h/mL)	6.09	2.34	1.31	466.40	287.78-755.90	179.01	110.45-290.13
AUC 0-0.58 (μg/h/mL)	8.99	3.72	2.30	390.44	251.21-606.85	161.71	104.04-251.34
AUC 0-0.67 (μg/h/mL)	12.08	5.39	3.52	343.55	227.18-519.53	153.34	101.40-231.89
AUC 0-0.83 (μg/h/mL)	18.03	9.24	6.36	283.49	195.09-411.95	145.21	99.93-211.00
AUC 0-1.00 (μg/h/mL)	23.71	13.65	9.54	248.48	176.57-349.68	143.08	101.67-201.35

There were no serious adverse events and no withdrawals from the study because of an adverse event. Three individuals reported a total of four adverse events, three of which were considered mild and one moderate, but none was considered to be related to the study drugs.

## Discussion

This study showed that the T_max _for the sodium ibuprofen formulation was less than half that of the standard ibuprofen acid formulation, suggesting that the rate of absorption of sodium ibuprofen was twice as fast as that of standard ibuprofen tablets. As expected, this increased rate of absorption was accompanied by an increase in the peak plasma concentration of ibuprofen. Furthermore, the CI of the ratio of the sodium ibuprofen/standard ibuprofen tablets for C_max _was outside the 80-125% limit specified in the Committee for Proprietary Medicinal Products (CPMP) guidelines [20] and the United States Food and Drug Administration (US FDA) guidelines for bioavailability and bioequivalence [21]. The fact that the sodium ibuprofen formulation was not bioequivalent with the standard ibuprofen acid formulation was expected given the faster absorption of ibuprofen from the sodium formulation. This is likely to be due to faster dissolution and more rapid availability of ibuprofen particles for absorption, as suggested by Sorgel *et al*. [9].

The literature suggests that the addition of poloxamer 407 to the ibuprofen acid formulation might have resulted in a faster absorption of ibuprofen than from standard ibuprofen formulation [15]. Although the time to reach the peak plasma concentration was, on average, 20 minutes faster with the ibuprofen/poloxamer tablets, this difference failed to achieve statistical significance. As it had been anticipated that the difference in T_max _between the ibuprofen acid and ibuprofen/poloxamer formulations would be 40 minutes, the study was not sufficiently powered (i.e. 31% only) for the observed difference to be statistically significant. The CI of the ratio of the ibuprofen/poloxamer to the standard ibuprofen tablets for C_max _was within the 80-125% limit specified in the CPMP guidelines [20] and FDA guidelines [21]. As the CI of the ratio of the ibuprofen/poloxamer to the standard ibuprofen tablets for AUC also fell within this limit, the ibuprofen/poloxamer and standard ibuprofen formulations can be considered bioequivalent.

The differences between the sodium ibuprofen formulation and the standard ibuprofen formulation would not be expected to result in adverse clinical consequences in terms of either safety or efficacy. In support of this, there were no clinically significant safety findings in the present study.

The data from this study show that the overall amount of drug to which the participants were exposed was the same for the three formulations. However, because of the faster absorption of ibuprofen from the sodium ibuprofen formulation compared with the standard formulation, it is expected that an OTC dose of two 256 mg sodium ibuprofen tablets may provide faster onset of analgesia without compromising safety or duration of analgesia. Previous studies have demonstrated that the median time to meaningful pain relief for ibuprofen arginate was half that of the standard ibuprofen [6,13,14]. Because the T_max _for sodium ibuprofen in this study (median: 35 min) was found to be similar to ibuprofen arginate (mean: 33.6 min) [9], it can be expected that sodium ibuprofen would induce analgesic effects with a similar onset of action as ibuprofen arginate. Such a formulation would be a valuable addition to the OTC choice currently available to individuals with acute pain. To this extent, a clinical trial was conducted to explore this and data are published separately [22].

## Conclusion

The sodium ibuprofen formulation is equivalent to standard ibuprofen in terms of extent of absorption, but its rate of absorption, as measured by T_max_, is significantly faster (i.e. it was absorbed twice as fast as standard ibuprofen) and C_max _is significantly higher. However, the ibuprofen/poloxamer formulation was bioequivalent to the standard ibuprofen formulation. Therefore, it is expected that an OTC dose of two 256 mg sodium ibuprofen tablets may provide faster onset of analgesic benefit, without compromising safety.

## Competing interests

This study was funded by Reckitt Benckiser Group plc. The Principal Investigator, Peter Dewland, was Medical Director at Simbec Research, UK. Sandie Reader and Phillip Berry are employees of Reckitt Benckiser Group plc.

## Authors' contributions

PD was the Principal Investigator and was responsible for the conduct of the study at Simbec. SR was the Clinical Project Manager at Reckitt Benckiser Healthcare (UK) and was the primary contact between Simbec and the sponsor. PB is the Global Medical Director at Reckitt Benckiser Healthcare UK. All authors read and approved the final manuscript.

## Pre-publication history

The pre-publication history for this paper can be accessed here:

http://www.biomedcentral.com/1472-6904/9/19/prepub
